# A generalizability study of the medical judgment vignettes interview to assess students' noncognitive attributes for medical school

**DOI:** 10.1186/1472-6920-8-58

**Published:** 2008-12-10

**Authors:** Tyrone Donnon, Elizabeth Oddone Paolucci

**Affiliations:** 1 Department of Community Health Sciences, Faculty of Medicine, University of Calgary, Calgary, Canada; 2 Department of Surgery, Faculty of Medicine, University of Calgary, Calgary, Canada

## Abstract

**Background:**

Although the reliability of admission interviews has been improved through the use of objective and structured approaches, there still remains the issue of identifying and measuring relevant attributes or noncognitive domains of interest. In this present study, we use generalizability theory to determine the estimated variance associated with participants, judges and stations from a semi-structured, Medical Judgment Vignettes interview used as part of an initiative to improve the reliability and content validity of the interview process used in the selection of students for medical school.

**Methods:**

A three station, Medical Judgment Vignettes interview was conducted with 29 participants and scored independently by two judges on a well-defined 5-point rubric. Generalizability Theory provides a method for estimating the variability of a number of facets. In the present study each judge (*j*) rated each participant (*p*) on all three Medical Judgment Vignette stations (*s*). A two-facet crossed designed generalizability study was used to determine the optimal number of stations and judges to achieve a 0.80 reliability coefficient.

**Results:**

The results of the generalizability analysis showed that a three station, two judge Medical Judgment Vignettes interview results in a G coefficient of 0.70. As shown by the adjusted *Eρ*^2 ^scores, since interviewer variability is negligible, increasing the number of judges from two to three does not improve the generalizability coefficient. Increasing the number of stations, however, does have a substantial influence on the overall dependability of this measurement. In a decision study analysis, increasing the number of stations to six with a single judge at each station results in a G coefficient of 0.81.

**Conclusion:**

The Medical Judgment Vignettes interview provides a reliable approach to the assessment of candidates' noncognitive attributes for medical school. The high inter-rater reliability is attributed to the greater objectivity achieved through the used of the semi-structured interview format and clearly defined scoring rubric created for each of the judgment vignettes. Despite the relatively high generalizability coefficient obtained for only three stations, future research should further explore the reliability, and equally importantly, the validity of the vignettes with a large group of candidates applying for medical school.

## Background

With an increased demand for accountability about which candidates to admit to medical school and the continuing growth in the number of qualified applicants, the president of the Association of American Medical Colleges (AAMC) expressed serious concern that the truly "compelling" personal characteristics of individual applicants are rejected for minor blemishes in their academic record [1]. The increasing importance of developing better methods of assessing candidates' personal attributes for admission into medical school was recently reviewed by Albanese et al. [2]. While professionalism in medicine is noted as the cornerstone of medical practice and underlines the behaviours expected of all doctors, the initial selection of medical students based on these characteristics is the first step in ensuring that future doctors manifest these attributes in practice.

Although the reliability of the interview has been improved through the use of structured approaches [3,4], there still remains the issue of identifying and measuring relevant attributes or noncognitive domains of interest. One of the major criticisms of the interview process has been that of content specificity, as there still seems to be disagreement about which of the compelling personal characteristics to measure [5]. In a move by the AAMC to establish consistent medical school objectives that meet society's expectations of physicians, a consensus was reached among leaders of 14 countries regarding the attributes that new doctors need to practice medicine [6]. Of the four principal attributes identified, the first (physicians must be altruistic) is related entirely to the promotion of specific altruistic, noncognitive characteristics and the fourth (physicians must be dutiful) emphasizes the importance of enhancing the ability to work collaboratively with other healthcare professionals and develop strong interpersonal skills.

### The Medical Judgment Vignette and Scoring Rubric

In an attempt to address the content specificity of the interview process and to better reflect the practice of medicine, we identified three areas of focus for the development of the Medical Judgment Vignettes: 1) major ethical dilemmas in medicine (moral), 2) relationships with patients and their families (altruistic), and 3) collaboration and clarification with staff and colleagues (dutifulness). In each scenario, the vignettes and probing questions were written and presented to the candidate in the third person. With each of the 3 to 4 probing questions posed to the candidate, a personal judgment of how the attending physician should respond was obtained by the interviewer. For example, in the 'moral' vignette a physician was involved indirectly with an assisted suicide of a patient suffering from Amyotrophic Lateral Sclerosis (ALS). Candidates were then asked to respond to the following question: "Should the doctor lose his license and, hence, ability to practice medicine?"

In the development of the vignettes, a group of experts from the medical school identified several main topics and related categories. Under the main heading of 'Moral/Ethical Dilemmas in Medicine', for example, 'Beginning of Life', 'Genetics', and 'End of Life' were three subheadings, each with further sub-themes identified such as 'abortions' and 'immunizations', 'stem cell research' and 'cloning', and 'euthanasia', respectively. As a key component of the Medical Judgment Vignettes interview, judges were trained in the protocol use for the semi-structured interview process. In particular, an objective approach in the presentation of the vignettes and probing questions was maintained throughout the process and with each of the candidates. Clarification of meaning through re-iteration of candidates' responses or asking for further elaboration on short "yes" or "no" answers became a main function of the interviewer in the semi-structured interview process.

In comparison with other admission's interview approaches, the Medical Judgment Vignettes are most similar to the Multiple Mini-Interview (MMI) in that a sequence of structured encounters are used in much the same way Objective Structured Clinical Exams (OSCE) stations are used in the assessment of clinical performance skills [7,8]. Unlike the MMI, however, in the Medical Judgment Vignettes we conceptualized each of the vignette measures within the context of medical practice and from the perspective of the attending physician. While this provides face validity to the interview process for both candidate and interviewer, it may also provide less biased responses from test-wise candidates, as the noncognitive attribute being measured is not stated explicitly in the vignette (as it is in the MMI). Candidates, therefore, are less likely to give socially desirable responses. In the 'altruistic' vignette, for example, the respondent may interpret the physician's interaction with a teenage cancer patient's and her mother's rejection of chemotherapy treatment for alternative non-medical therapies as an ethical or moral dilemma. While taking care to avoid potential biases that may favour candidates with previous clinical knowledge, the focus of the Medical Judgment Vignettes interview is on a domain specific to the noncognitive attribute in question (e.g., moral, altruistic, dutifulness).

The Medical Judgment Vignettes are scored on a well-defined rubric based on Colby and Kohlberg's work on moral reasoning as functional stages of development [9]. According to Kohlberg's theory of moral development, people will proceed through stages of moral reasoning as they mature (Table 1). Although an individual will vary in their rate of progression and the end stage obtained, the ordering of the stages is consistent. In the process of assigning a stage score, the logic of the reasoning or the justification provided is considered rather than a specific set of moral beliefs or value. Kohlberg's scale on moral development has been validated across many socio-cultural situations and shown to have applicability in the context of medical education [10-14].

**Table 1:** Overview of Kohlberg's stages of moral development

**Preconventional Level**	
*Stage 1*: Obedience and Punishment Orientation	Focus on avoidance of punishment by not breaking the rules and deference to authority figures.
*Stage 2*: Individualism and Exchange Orientation	Acceptance of alternative views as right and wrong is determined by what satisfies the individual's particular needs.
**Conventional Level**	
*Stage 3*: Good Interpersonal Relationships Orientation	Meet the expectations of what is right because people expect it as part of mutual interpersonal relationships.
*Stage 4*: Maintaining Social Order Orientation	Emphasis is on obeying social order, respecting the dignity of all while conforming to the laws of the group or institution.

**Postconventional Level**	
*Stage 5*: Balance of Social Contract and Individual Rights Orientation	Conceptualize society in a theoretical manner, stepping back from existing society and considering the relativity of group and individual values with respect to what society ought to uphold.
*Stage 6*: Universal Ethical Principles	Defined by universal moral principles (what a society should uphold) and sense of personal commitment to them.

In assessing the participant's capacity for reasoning, a value is assigned to an individual if the frequency of the responses is predominantly at that stage of development. Candidates score at preconventional stages of development when responses to the 'moral' and 'altruistic' vignettes fail to move beyond stage 1 – "physician's actions focus on avoidance of punishment" and stage 2 – "physician's interactions with patient and family reflect acceptance, but are indifferent to the interpersonal relationship with the doctor", respectively (see Table 2 Excerpts). At the conventional level, stage 3 focuses on the "physician having a 'good' interpersonal relationship orientation" and stage 4 on the "physician's ability to maintain a social order orientation." To achieve at the highest postconventional level, the respondent must emphasize the role the physician plays in a good and just society where stage 5 represents the "physician's balance of social contract and individual rights orientation." Stage 6 was removed as a general measure of development as this stage reflects decisions of conscience, based on self-chosen ethical principles appealing to universality and associated with moral leaders such as Gandhi and the Dali Lama. Probing questions were designed to provide candidates with an opportunity to reason through various aspects of the medical dilemma related to the Medical Judgment Vignette. The structured scoring rubric provides objectivity in scoring candidates' performance by anchoring applicable responses to clearly defined stages established *a priori*.

**Table 2:** Excerpts of various responses to the 'Moral' Medical Judgment Vignette

**Preconventional Level**	
Stage 1: Obedience and Punishment Orientation	"I just don't think that it's a doctor's position to help somebody to die. It's against their ethical and legal responsibilities and to their patient...it would depend on what the rules and regulations of the governing bodies are..."
Stage 2: Individualism and Exchange Orientation	"So, from a certain standpoint I do agree, because it's not the doctor that's really making the decision, he's just kind of complying with the patient's request...As long as he doesn't go around willy-nilly recommending euthanasia to a whole bunch of people with diseases like this. As long as he discusses the implications with the patient and the different possibilities that are available for things and the patient is well-aware of all the implications of the decisions."
**Conventional Level**	
Stage 3: Good Interpersonal Relationships Orientation	"Yes, I think you have to. If he has known her for 15 years he probably has quite a good relation with her and knows she truly wants to die. I don't know what the legal ramifications are yet in medicine, but there's quality of life and length of life issues and I think it's in this case, for sure that she has a right to die and if you can make her more comfortable, even though he is a medical doctor, I think that's totally appropriate."
Stage 4: Maintaining Social Order Orientation	"No. I don't. I think that end of life palliative care is a very touchy subject, but I believe that most elderly folk, although they do express a wish to die at home, if they are supported by a family system and a social system that is adequate for their needs at that time, there should be no reason to introduce the idea of euthanasia."

**Postconventional Level**	
Stage 5: Balance of Social Contract and Individual Rights Orientation	"The right decision has multiple dimensions in that there are legal, moral, ethical aspects of whether it is right or wrong...I believe from a legal standpoint that he is wrong in his decision. Morally and ethically, I believe the decision should be left to the individual and if her decision was made at a point in her life where she was of sound mind and had received appropriate counselling from her physician, her family, children, relatives, and if they had explored and openly communicated her desire to terminate her life at whatever time she wanted to, then it would be the right decision."

Although the Medical Judgment Vignettes has been shown to have good predictive power on noncognitive clinical performance measures in clerkship [15], questions of reliability and feasibility for use of the vignettes for the medical school admission's process remain. Accordingly, the main purpose of the present generalizability study was to examine the reliability of the semi-structured, Medical Judgment Vignette interview approach for assessing noncognitive attributes (i.e., moral, altruistic, and dutiful) contextualized within the costly and high stakes setting of interview selections for medical school.

## Methods

### Participants and procedures

A total of 29 first year medical students participated voluntarily in a 15 to 20 minute semi-structured interview. The sample consisted of 18 females (62%) and 11 males (38%) with a mean age of 26.7 years (*SD *= 4.1; range 19 to 37). The semi-structured interview process was conducted by a trained counselling psychologist and consisted of reading aloud each Medical Judgment Vignette while the participant followed along with his or her own printed copy. Responses to the open-ended probing questions for all 29 students were tape-recorded, transcribed and scored independently by TD and EOP on the 5-stage moral, altruistic, and dutiful scoring rubric. This study is in compliance with the Helsinki Declaration, was approved by the Conjoint Health Research Ethics Board of the University of Calgary and signed consent was obtained by all participants.

### Generalizability Theory

Generalizability theory provides a method for estimating the variability of a number of facets. For example, in the present study each judge (*j*) rated each participant (*p*) on all three Medical Judgment Vignette stations (*s*). In this two-facet fully crossed research design, an analysis of variance (ANOVA) was used to estimate the variability of students' scored performance as each variance component defined may contribute to error in measurement. These consist of the three main effects (participants, judges, stations), the three two-way interactions between main effects (*p *× *j*, *p *× *s*, *j *× *s*) and the three-way interaction effect (*p *× *j *× *s*) that is confounded with random error (*e*) as a function of the crossed design. Like a reliability coefficient that ranges from 0 to 1.0, a generalizability coefficient can be interpreted as an index of the dependability of a particular measurement process.

## Results

The participants were representative of their class (2007) by both sex (60% females and 40% males) and age (*M *= 25.6 years, *SD *= 4.3); *p *> .05. The mean interrater reliability coefficient between the two independent judges was found to be Kappa = 0.95 across the three Medical Judgment Vignettes. Although students' performance on all three stations covered the full range of potential scores across the five stages, the mean scores for all three vignettes were between stages 2 and 3: 1) major ethical dilemmas in medicine (*M *= 2.6, *SD *= 1.4), 2) relationships with patients and their families (*M *= 2.5, *SD *= 1.1), and 3) collaboration and clarification with staff and colleagues (*M *= 2.5, *SD *= 1.4). Correlations between the vignettes ranged from *r *= 0.22 (*p *= 0.25) between stations 1 and 2, to *r *= .43 (*p *< .05) between stations 1 and 3, and *r *= .49 (*p *< .01) between stations 2 and 3. No significant differences were found by sex or age on students' performance scores for the three Medical Judgment Vignettes.

### Reliability analysis

In this two-facet crossed design (*c *× *j *× *s*), ANOVA was used to calculate the variance associated with each of the seven components. As shown in Table 3, the majority of the variance explained was from the students themselves (41.7%) and the two-way interaction effect between the students and the Medical Judgment Vignette stations (51.1%). All other main and two-way interactions between these effects were negligible. The three-way interaction confounded with other random error not accounted for in this generalizability study, however, did result in 6.8% of the variance explained.

**Table 3:** Variance component results of a 29 participants, two judges and three Medical Judgment Vignettes stations

**Source of Variation**	**df**	**Mean Squares**	**Variance Component**	**Variance Explained**
**Participants (*p*)**	28	6.597	0.765	41.69
**Judges (*j*)**	1	0.508	0.003	0.16
**MJV Stations (*s*)**	2	0.089	-0.031	0.00
***p × j***	28	0.118	-0.002	0.00
***p × s***	56	2.001	0.938	51.12
***j × s***	2	0.227	0.004	0.22
***pjs, e***	56	0.125	0.125	6.81
Total				100.00

Note: negative variance component estimates may occur – in this present study, the reason for their occurrence is that the true value of the variance equals zero.

In determining the generalizability coefficient (*Eρ*^2^), the variance components are used as sample estimates to determine relative decisions about the students' performance. In this case, with two judges (*n*_*j *_= 2) and three vignettes (*n*_*s *_= 3) we found the generalizability coefficient to be *Eρ*^2 ^= 0.70. To obtain an optimal level of generalizability for making decisions about the future use of the Medical Judgment Vignettes, we used the formula noted in Table 4 to explore the advantages of adding or reducing the number of judges or stations used in the interview process. As shown by the adjusted *Eρ*^2 ^scores, since interviewer variability is negligible, increasing the number of judges from two to three does not improve the generalizability coefficient. Increasing the number of stations, however, does have a substantial influence on the overall dependability of this measurement. Increasing the number of stations to six while reducing the number of raters to a single judge, for example, results in a generalizability coefficient of *Eρ*^2 ^= 0.81.

**Table 4:** Generalizability study results of a three station Medical Judgment Vignettes semi-structured interview

**Medical Judgment Vignette Stations & Judge Interviewers Combinations**	***n *(stations)**	***n *(judges)**	*Ep^2^
**1 MJV station, 5 judges**	1	5	0.44
**2 MJV stations, 4 judges**	2	4	0.60
**3 MJV stations, 3 judges**	3	3	0.70
**3 MJV stations, 2 judges**	3	2	0.70
**4 MJV stations, 2 judges**	4	2	0.75
**5 MJV stations, 1 judge**	5	1	0.78
**6 MJV stations, 1 judge**	6	1	0.81

*Note: $E\rho_{\delta }^{2}=\frac{\sigma_{c}^{2}}{\sigma_{c}^{2}+\left[\frac{\sigma_{cs}^{2}}{n_{s}}+\frac{\sigma_{cj}^{2}}{n_{j}}+\frac{\sigma_{cjs}^{2}}{n_{s}n_{j}}\right]}$; where *c *= candidates, *s *= stations, *j *= judges

## Discussion

The main findings of the present study are that: 1) the Medical Judgment Vignettes interview had high reliability as an assessment of students' noncognitive attributes, 2) increasing the number of vignettes (i.e., stations) will increase the overall reliability of the interview process, and 3) a semi-structured interview format with a clearly defined scoring rubric resulted in high inter-rater reliability and reduced the need for multiple judges at each station.

Although the goal of using the Medical Judgment Vignettes interview approach is multi-dimensional, one of its main advantages is that it establishes a semi-structured interview format with pre-determined, open-ended questions that are asked consistently of all participants. Open-ended questions provide an opportunity for the respondent to introduce relevant information, personal ideas and conceptual understandings that the interviewer or judge may not have thought of during the question selection. The use of the stages scoring rubric, however, is an essential component of the medical judgment rating process. In particular, the potential subjectivity of a participant's response to the probing questions is quantified by the interviewer or judge a priori through an understanding of how each stage of response is anchored to a respective stage in the pre- to post-conventional judgment criteria. In further support of the development of the scoring rubric for each of the vignettes, the generalizability analysis showed that the amount of variance between judges was trivial (0.16%) compared to the total variance explained. Further empirical studies of the reliability of these vignettes, however, are required.

As explained earlier, generalizability theory provides a method to determine the dependability of an assessment approach by isolating the main and interaction effects that can lead to sources of measurement error. In determining how many conditions of each facet are needed in the future to achieve an optimal level of generalizability (e.g., greater or equal to 0.80) a Decision study analysis was completed for various numbers of judges and stations. We determined that decisions about the Medical Judgment Vignettes interview process would support an increase in number of stations without an increase in judges. In particular, to achieve a generalizability coefficient of 0.81 the recommendation is to reduce the number of raters to a single judge while increasing the number of vignettes or stations to six. Similar findings by Eva et al. [7] have found that a G-coefficient of 0.81 can be obtained with the MMI using 6 stations with 2 interviewers at each station. The 'interviewer within station' estimated variance, however, was found to be substantial (accounting for 21% of the variance) and in order to maintain high reliability above 0.80 with a single judge, as many as 12 stations would be required.

Although both the reliability and validity of the Medical Judgment Vignettes interview appears promising, the selection of participants for convenience and limited sample size used in this study needs to be addressed in subsequent research. With the inclusion of additional stations and the expansion of potential measures (e.g., collegiality, compassion, empathy, honesty, etc.), the need for guidance in scenario design and scoring rubric development will be important if similar success with this interview format is to be obtained. A number of practical issues related to the training of interviewers in the understanding and purpose of the Medical Judgment Vignette interview approach will also be essential for success when administered to a large number of candidates. In particular, consistency in the use of the scoring rubric as it pertains to each of the vignettes developed will be an important component to the maintenance of low inter-rater variable and, hence, error of measurement. Although the scoring rubric provides a clear anchoring for marking the Medical Judgment Vignettes, the authors were well grounded in the use of the rubric and future examiners may not have the same depth of understanding or time needed to train them appropriately. In particular, the use of a detailed scoring rubric may reduce the variability between raters, but the time required to educate the examiners in its use may simply be too prohibitive.

## Conclusion

In general, the Medical Judgment Vignettes provide face validity to the medical school admission's interview process. Presented as tangible, third-person physician encounters, the undifferentiated vignettes allow individuals to frame their judgments about actions in a non-threatening and personalized manner. The high inter-rater reliability is attributed to the greater objectivity achieved through the use of the semi-structured interview format and the clearly defined scoring rubric created for each of the judgment vignettes. Despite the relatively high generalizability coefficient obtained for only three stations, future research should further explore the reliability, and equally importantly, the validity of the vignettes with large group of candidates for medical school. Although the predictive validity of the Medical Judgment Vignettes has been shown to have moderate effect size measures (*r *= .45) across clinical performance measures in clerkship [15], further research on how well these noncognitive outcomes reflect physician practice in residency and beyond is needed.

## Competing interests

The authors declare that they have no competing interests.

## Authors' contributions

All authors made substantial contributions to the conception and design of the innovations outlined in the paper, the acquisition of the data and the interpretation of the results of the analysis. All authors helped in revising the draft critically for important intellectual content. All authors approved the final version of the paper prior to submission. In addition TD was responsible for the analysis of the data and wrote the first draft of the paper.

## Pre-publication history

The pre-publication history for this paper can be accessed here:


